# BRAD, the genetics and genomics database for Brassica plants

**DOI:** 10.1186/1471-2229-11-136

**Published:** 2011-10-13

**Authors:** Feng Cheng, Shengyi Liu, Jian Wu, Lu Fang, Silong Sun, Bo Liu, Pingxia Li, Wei Hua, Xiaowu Wang

**Affiliations:** 1 Institute of Vegetables and Flowers, Chinese Academy of Agricultural Sciences, Beijing, 100081, China; 2 The Oil Crops Research Institute, Chinese Academy of Agricultural Sciences, Wuhan, 430062, China

## Abstract

**Background:**

Brassica species include both vegetable and oilseed crops, which are very important to the daily life of common human beings. Meanwhile, the Brassica species represent an excellent system for studying numerous aspects of plant biology, specifically for the analysis of genome evolution following polyploidy, so it is also very important for scientific research. Now, the genome of *Brassica rapa *has already been assembled, it is the time to do deep mining of the genome data.

**Description:**

BRAD, the Brassica database, is a web-based resource focusing on genome scale genetic and genomic data for important Brassica crops. BRAD was built based on the first whole genome sequence and on further data analysis of the Brassica A genome species, *Brassica rapa *(Chiifu-401-42). It provides datasets, such as the complete genome sequence of *B. rapa*, which was *de novo *assembled from Illumina GA II short reads and from BAC clone sequences, predicted genes and associated annotations, non coding RNAs, transposable elements (TE), *B. rapa *genes' orthologous to those in *A. thaliana*, as well as genetic markers and linkage maps. BRAD offers useful searching and data mining tools, including search across annotation datasets, search for syntenic or non-syntenic orthologs, and to search the flanking regions of a certain target, as well as the tools of BLAST and Gbrowse. BRAD allows users to enter almost any kind of information, such as a *B. rapa *or *A. thaliana *gene ID, physical position or genetic marker.

**Conclusion:**

BRAD, a new database which focuses on the genetics and genomics of the Brassica plants has been developed, it aims at helping scientists and breeders to fully and efficiently use the information of genome data of Brassica plants. BRAD will be continuously updated and can be accessed through http://brassicadb.org.

## Background

Brassica species belong to the Brassicaceae family, which contains about 3700 species from 338 genera, including the widely studied model plant *Arabidopsis thaliana*. Brassica species include both vegetable and oilseed crops that contribute about 10% of the world's vegetable production and about 12% of world's edible vegetable oil production [1,2]. The diploid genomes of the six widely cultivated Brassica species are described by the famous "U's triangle" (genome A, B, C, AB, BC and AC, corresponding to *B. rapa*, *B. oleracea*, *B. nigra*, *B. juncea*, *B. napus*, and *B. carinata*, respectively [3]. The A genome species, *B. rapa*, is a major vegetable and also an oil crop in Asia and Europe. Because of their importance as crops and as models to study complex genome hybridization and polyploidization [4,5], genetic and genomic research on Brassicas has intensified over recent years, generating ever increasing sets of data, such as Brassica genome sequences, genetic markers, expressed sequence tags (ESTs) and quantitative trait loci (QTLs).

The recently completed initial assembly of the whole genome sequence of the *B. rapa *cultivar line 'Chiifu-401' is now available [6]. Based on the needs of the Brassica research community and on the distribution of the bulk genomic data, BRAD has been built. It was developed as an important repository for whole genome scale genetic and genomic data and for related resources of Brassica crops. BRAD was also designed as an initial access point for other related web pages and specialized datasets. It now provides datasets of the Brassica A genome (*B. rapa*, Chiifu-401), including the *de novo *assembled genome sequence derived from second-generation sequencing technologies and from BAC end sequences, predicted genes, associated annotations (InterPro, KEGG2, SwissProt), as well as genetic markers and maps of *B. rapa*.

In this article we present an overview of the major sections of BRAD, and introduce a keyword searching tool that we have developed and the tools of BLAST and Gbrowse that enable data mining in BRAD.

## Construction and Content

**Figure 1:**
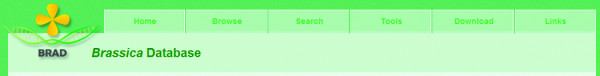
**Navigating BRAD**. There are four major sections: Browse, Search, Tools, and Resources (Download and Links). Moving the cursor over tabs will activate the pull-down menus, which will lead users directly to the specific pages in BRAD.

With the analysis of the first available genome sequence of B. rapa, We developed BRAD, the Brassica database. There are four major sections in BRAD (Figure 1): Browse, Search, Tools and Resources.

### Browse

In this section, BRAD provides 1, 160 genetic markers from three population lines of *B. rapa: *RCZ16_DH, JWF3P, and VCS_DH. These markers, including 758 SSR and 402 InDel markers, cover all ten chromosomes [7,8]. RCZ16_DH is a population developed from a cross between a rapid cycling line, L144, and a summer type Chinese cabbage double haploid (DH) line, Z16 [9]. There are 119 DH lines in this population. Markers of RCZ16_DH were developed based on resequence data of the parents L144 and Z16. By aligning the resequence data to the assembled genome of Chiifu-401, we obtained 26, 693 InDel markers between L144 and Z16, of which 402 markers were used to anchor the *de novo *assembled scaffolds to the 10 chromosomes. The other two maps, JWF3P and VCS_DH, were integrated from the public database http://www.brassica-rapa.org to offer users more options.

### Search

This section was developed to annotate predicted genes and to help users locate specific genes in *B. rapa*. Totally, there are 41, 174 genes predicted in genome of *B. rapa*. There are slightly less CDS for each gene in *B. rapa *when comparing to that of *A. thaliana*, while the size of each intron of *B. rapa *is a little bigger than that of *A. thaliana *(Table 1). It may indicate that paralogous genes generated by genome triplication in *B. rapa *were differentiated [6], some coding exons were lost in this process and enlarged the average size of introns in *B. rapa*. There are three sub-categories in search part: searching using annotations, syntenic genes, and flanking regions.

**Table 1:** The comparison of genes between *B. rapa *and *A. thaliana*.

	#Gene	#CDS/gene	CDS size	Gene size	Intron size
***B. rapa***	41, 174	5.03	233.04	1171.56	1077.31
***A. thaliana***	27, 379	5.38	224.70	1209.13	880.30

#### 1) **Annotations**

There are six annotation datasets collected here: Swissprot annotation, Trembl annotation, KEGG annotation, InterPro domain annotation, Gene Ontology and the BLASTX (best hit) of *B. rapa *to *A. thaliana*. Swissprot and Trembl annotations are generated by BLASTP best hit (cutoff E-value: 1e-5) of predicted *B. rapa *proteins in the Swiss-Prot and TrEMBL databases; *B. rapa *genes are then mapped to KEGG pathway maps based on the best hit from the Swiss-Prot database; InterPro is used to annotate motifs and domains in *B. rapa *genes by comparison to public databases, including Pfam, PRINTS, PROSITE, ProDom and SMART using applications hmmpfam, fprintscan, ScanRegExp profilescan, blastprodom, and hmmsmart. Gene Ontology information is extracted from the InterPro results. We also use orthologous genes between *B. rapa *and the model plant *A. thaliana *to annotate *B. rapa *genes. These datasets are used to annotate predicted genes according to different aspects, such as nucleotide sequences, proteins and domains.

#### 2) **Orthologous genes**

Syntenic and non-syntenic orthologs between *A. thaliana *and *B. rapa *were provided in BRAD to help users to link *B. rapa *gene information to that of the well studied model plant *A. thaliana*.

BRAD presents a set of genes that show conserved synteny between *A. thaliana *and the three subgenomes of *B. rapa *(the three subgenomes originated from genome triplication), and that are listed according to the genes' order in *A. thaliana*. We determined a gene-pair to be in synteny not only by their sequence homozygosity but also by the homozygosity of their flanking genes. With this rule, 30, 773 syntenic pairs between *B. rapa *and *A. thaliana *were obtained, and there were 9, 293, 6, 683 and 2, 346 *A. thaliana *genes which have 1, 2 and 3 paralogous copies in the *B. rapa*'s subgenomes LF, MF1, and MF2, respectively. LF, MF1 and MF2 are abbreviations for less fractionized, more fractionized 1 and more fractionized 2, respectively, denoting subgenomes with more or fewer genes retained. We separated the three subgenomes according to comparative analysis with the *A. thaliana *genome and then with respect to both gene orders and gene densities of the subgenomes [6].

Non-syntenic genes between *A. thaliana *and *B. rapa *were determined under two rules. First, the parameters of BLASTP alignment should be satisfied: identity > 70%, coverage of *A. thaliana *gene > 75%, coverage of *B. rapa *gene > 75%. Second, two genes from an orthologous pair should not be syntenic genes. Totally, there were 17, 159 such non-syntenic orthologous pairs determined.

#### 3) **Flanking region searching**

This section was developed to help users find genomic elements that are co-located with or that flank a region of interest. Users can input a physical position, for example of a gene ID or genetic marker, to perform the search. All the genomic features, such as genes, transposons, RNAs (miRNA, tRNA, rRNA and snRNA) that are located near the searched region are collected and displayed in a table. A link to Gbrowse provides an option to visualize the search region under the background of the chromosome. This is a useful tool for certain studies, such as the fine mapping of QTLs. Once QTLs have been obtained, markers can be aligned to the genome sequence with the BLAST tool to get the physical positions of the markers. The flanking region of these markers can then be searched to locate candidate genomic elements, such as genes or miRNAs, which might be the causal factors of the QTLs.

As research progresses, we will further enable the searching of flanking regions by adding more datasets, making it an integrative and valuable resource pool for molecular geneticists, breeders and all other researchers who are interested in Brassica plants.

### Tools

BLAST and Genome browse (Gbrowse) are embedded to help users mine and visualize the genome data.

#### 1) BLAST

We utilized the standard wwwblast modules to help users perform sequence analysis. BLAST databases, such as genome, gene and protein sequences of *B. rapa*, EST sequences of *B. rapa*, Brassicas, and Cruciferaes are provided here.

#### 2) Genome browse (Gbrowse)

We used the Genome Browser tools developed by the Generic Model Organism Database Project, http://gmod.org to visualize the genome of *B. rapa *[10]. Three major levels are displayed: genome segment, flanking region of the search area and the exact target. We now provide predicted genes, transposons, multiple types of RNA sets, genetic markers in Gbrowse.

### Resources

In addition to the Browse, search, and tools described above, BRAD provides bulk data downloads, including genome and gene sequences, gene annotations and other predicted genomic elements. In addition, BRAD makes numerous community resources available either as data or as website links. These include other websites of laboratories focusing on Brassicaceae, meetings of potential interest to Brassica researchers and collections of sites about Brassica breeding.

## Utility

### General guidelines for using BRAD

Browse genetic markers and maps.

Search using annotations and Syntenic genes.

Gbrowse: genome visulization.

For each marker in the part of Browse genetic markers and maps, we present its genetic and physical positions and primer information and the parental populations. Users can access these data in the Browse section by following order: chromosome selection → population specification → detailed marker information → click marker ID for primer information.

In section of search using annotations, users can find genes with functions of interest by submitting a keyword, such as flower or growth, then relevant records will be selected from the six annotation datasets as described above. Clicking on the selected records will then lead users to genes with annotations related to the keyword. A further click of the gene ID will provide users with more further information of this gene in BRAD.

**Figure 2:**
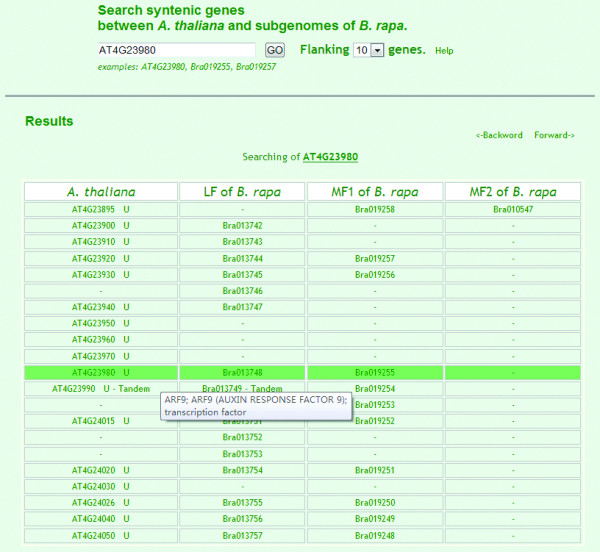
**Syntenic gene searching between *A. thaliana *and *B. rapa*'s three subgenomes**. Taking the *A. thaliana *gene AT4G23980 as an example, search results are presented in a table. The first column lists the *A. thaliana *gene IDs, followed by genomic blocks in the ancestral karyotype. Tandem genes are packed, and only the first gene of the tandem array is listed (AT4G23990) while the others can be obtained by clicking 'tandem'. The next three columns show genes from the subgenomes, LF, MF1 and MF2 of *B. rapa*. For each row, listed genes are in syntenic relationships. Moving the cursor over an *A. thaliana *gene gives a floating box containing the gene's annotation, while moving the cursor over a *B. rapa *gene produces the supporting information of its syntenic relationship to the gene in *A. thaliana*.

Syntenic genes can only be searched for using *A. thaliana *and *B. rapa *gene IDs. In the web of syntenic paralogs, the pull-down 'flanking' menu has two options (10 or 20), which means it can extend 10 or 20 genes up- and down-stream from the searched gene. In the tabulated output (Figure 2), the targeted gene is colored dark green. Each *A. thaliana *gene corresponds to 1, 2 or 3 genes in the *B. rapa *subgenomes. '-' indicates that no gene was identified. Moving the cursor over the ID of a gene expands the functional annotations of *A. thaliana *genes and the detailed supporting information of synteny relationships of *B. rapa *genes to that of *A. thaliana*.

**Figure 3:**
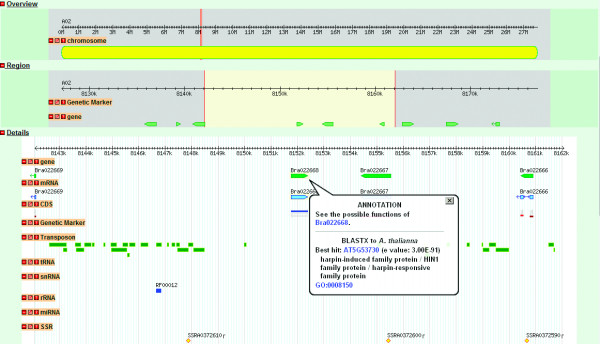
**Genome sequence view in Gbrowse of a region in chromosome A02 of *B. rapa***. The tracks shown in the detailed section are gene models from the 1.01 genome version of *B. rapa *(*B. rapa *Genome Sequencing Project) and indicate mRNA, CDS, genetic marker, TEprotein, Transposon, miRNA, tRNA, snRNA, rRNA and SSR. For the gene model track, a click on a gene provides a contextual menu with relevant links to the gene's annotations and to its best hit gene (BLASTX) in *A. thaliana *accompanied by its annotation text. For other tracks, a click on a feature leads users to its detailed annotation and sequence information.

The Gbrowse visualizes functional elements (genes, non-coding RNAs, TEs, genetic markers) of the genome of *B. rapa *under one frame, and we made links of genes in Gbrowse to the other applications in BRAD. By clicking a gene icon in Gbrowse, users can get the links of its annotation, the best BLASTX hit to *A. thaliana*, and the function and Gene Ontology (GO) of the matching gene, as shown in Figure 3.

### The search navigation

In order to help users to quickly access to all the information of an interested gene in BRAD, we embedded a javascript dialog window as navigation to each gene ID in the output tables of BRAD. Through combining the accesses of many datasets at one window, this navigation can lead users to different resources of the target genes, which facilitates the use of BRAD. There are two types of genes in BRAD now, genes of *B. rapa *and *A. thaliana*. For *B. rapa *gene, the navigation window integrates resources as the annotations, syntenic or non-syntenic orthologs, gene sequence, functional elements in gene's flanking regions and data visualization in Gbrowse, etc. For *A. thaliana *gene, navigation window provides links to resources of the syntenic or non-syntenic orthologs, annotations in BRAD and the TAIR databases.

## Discussions

A few databases of *Brassica rapa*, such as BrassEnsembl database http://www.brassica.info/BrassEnsembl/index.html, CropStore database http://www.cropstoredb.org/brassica/, and Brassica Genome Database http://www.plantgdb.org/BrGDB/, mainly focused on genome data dissemination (CropStore, Brassica Genome Database) and visualization (BrassEnsembl). BRAD was built to help users to mine data from the genome sequence of *Brassica rapa *easily and effectively, it had its own specific features and advantages when comparing to existing databases. First, BRAD made accurate and useful links from the bulk information of model plant *A. thaliana *to the newly assembled genome of *B. rapa *and offered detail annotation of *B. rapa *genes, it provided features as syntenic and non-syntenic orthologs between *A. thaliana *and *B. rapa*, main gene families in *B. rapa *according to that in *A. thaliana*, gene annotation information from multiple annotation databases (KEGG, InterPro, Swissprot, Tremble), etc. Second, BRAD was an initial genome data repository of *B. rapa*, other databases used or will use data in BRAD as basic data to develop their specific functions, we will improve and continuously update the assembled genome and release it in BRAD.

BRAD will include data sets of all Brassica plants (such as *B. oleracea*, *B. nigra *and *B. napus*) when they are available. In addition, new data will be processed first and then appropriately integrated or linked to the existing datasets. The data types listed below will soon be added to BRAD:

- browse of gene families, such as families of NBS genes, Auxin genes, Transcription factor genes, etc.

- allele data and frequencies of genetic markers generated from genome resequences of different lines of *B. rapa*.

- haplotypes (derived from SNPs mapping) of the *B. rapa *germplasm collection.

- levels of gene expression generated from transcriptome data in different organs of *B. rapa*.

- synteny browser of *B. rapa *to *B. oleracea*.

## Conclusions

BRAD, a new database which focuses on the genetics and genomics of the Brassica plants has been developed. Comparing with the existing database of Brassica plants, BRAD has its specific functions and advantages, specially for its annotations and deep mining of the recently assembled genome of *B. rapa*, as well as the use of the information from the model plant *A. thaliana*. Aimed at helping scientists and breeders to fully and efficiently use the information of genomics and genetics datasets of Brassica plants, BRAD will continuously improve its applications and integrate more available datasets in the future. We propose that BRAD will be a valuable resource for the scientists of comparative genomics, plant evolution, and molecular biology, and the breeders of Brassiceae.

## Availability and Requirements

Database name: BRAD

Database homepage: http://brassicadb.org

Browser requirement: the application is optimized for Internet Explorer. However, it also works well with Mozilla Firefox and Safari.

Datasets in BRAD are freely available. Please use the link 'Contact Us' on the BRAD homepage or email Dr. Xiaowu Wang wangxw@mail.caas.net.cn to request specific data subsets.

## Competing interests

The authors declare that they have no competing interests.

## Authors' contributions

XW and FC conceived the study. FC processed the data and developed the database. FC prepared the manuscript, XW and JW improved the manuscript. JW tested the web application and tools and provided feedback. LF maintained the database. SL, SS, BL, PL and WH prepared the basic datasets. All authors read and approved the final manuscript.
